# Assessing COVID-19 risk with temporal indices and geographically weighted ordinal logistic regression in US counties

**DOI:** 10.1371/journal.pone.0265673

**Published:** 2022-04-06

**Authors:** Vivian Yi-Ju Chen, Kiwoong Park, Feinuo Sun, Tse-Chuan Yang

**Affiliations:** 1 Department of Statistics, Tamkang University, Taipei, Taiwan; 2 Department of Sociology, The University of New Mexico, Albuquerque, NM, United States of America; 3 Global Aging and Community Initiative, Mount Saint Vincent University, Halifax, Nova Scotia, Canada; 4 Department of Preventive Medicine and Population Health, University of Texas Medical Branch, Galveston, TX, United States of America; Universidade Federal de Minas Gerais, BRAZIL

## Abstract

**Purpose:**

Research on the novel coronavirus diseases 2019 (COVID-19) mainly relies on cross-sectional data, but this approach fails to consider the temporal dimension of the pandemic. This study assesses three temporal dimensions of the COVID-19 infection risk in US counties, namely probability of occurrence, duration of the pandemic, and intensity of transmission, and investigate local patterns of the factors associated with these risks.

**Methods:**

Analyzing daily data between January 22 and September 11, 2020, we categorize the contiguous US counties into four risk groups—High-Risk, Moderate-Risk, Mild-Risk, and Low-Risk—and then apply both conventional (i.e., non-spatial) and geographically weighted (i.e., spatial) ordinal logistic regression model to understand the county-level factors raising the COVID-19 infection risk. The comparisons of various model fit diagnostics indicate that the spatial models better capture the associations between COVID-19 risk and other factors.

**Results:**

The key findings include (1) High- and Moderate-Risk counties are clustered in the Black Belt, the coastal areas, and Great Lakes regions. (2) Fragile labor markets (e.g., high percentages of unemployed and essential workers) and high housing inequality are associated with higher risks. (3) The Monte Carlo tests suggest that the associations between covariates and COVID-19 risk are spatially non-stationary. For example, counties in the northeastern region and Mississippi Valley experience a stronger impact of essential workers on COVID-19 risk than those in other regions, whereas the association between income ratio and COVID-19 risk is stronger in Texas and Louisiana.

**Conclusions:**

The COVID-19 infection risk levels differ greatly across the US and their associations with structural inequality and sociodemographic composition are spatially non-stationary, suggesting that the same stimulus may not lead to the same change in COVID-19 risk. Potential interventions to lower COVID-19 risk should adopt a place-based perspective.

## Introduction

The United States (US) has been the epicenter of the novel coronavirus disease 2019 (COVID-19) global pandemic since late January, 2020. The US population makes up 4 percent of the world population [1]; however, as of August 31, 2021, there are more than 38 million confirmed COVID-19 cases and over 635,000 deaths in the US [2], which account for approximately 18 percent of confirmed cases and 14 percent of COVID-19 deaths worldwide. While the pandemic has been contained with vaccination, the numbers of confirmed COVID-19 cases and deaths since late 2020 in the US have increased rapidly. Many scholars have paid attention to the health disparities in COVID-19 infections *within* the US, and this line of knowledge inquiry has documented disparities along several sociodemographic dimensions. For example, racial/ethnic minorities (e.g., non-Hispanic blacks and Hispanics) carry a disproportionate burden of COVID-19 infections and deaths in contrast to non-Hispanic whites [3–5]. High levels of residential segregation between non-Hispanic whites and other racial/ethnic minorities further aggravate the burden among minorities [6–8]. Moreover, areas with low socioeconomic status (e.g., low median income and educational attainment) observe higher COVID-19 case and mortality rates [9, 10], and counties with high concentrations of older adults recover from the pandemic more slowly compared with those with low proportions of older adults [11].

Beyond these sociodemographic disparities in COVID-19 outcomes, there is a growing interest in the spatial patterns of COVID-19 cases and deaths in the US. Sun and colleagues [12] apply various spatial econometrics models to the data of contiguous US counties and find that counties with high COVID-19 prevalence rates cluster in both coasts and the Black Belt. Using clustering analysis, Andersen et al. [13] suggest that counties in the northeastern, southeastern, and southwestern region of the US tend to have higher COVID-19 case and mortality rates than counties in other regions. To account for spatial non-stationarity in the determinants of COVID-19, geographically weighted regression (GWR) modeling techniques have been employed recently. For example, Karaye et al. [14] employ GWR to explore spatial variability in the impacts of social vulnerability on COVID-19 case counts across US counties. Mollalo et al. [15] adopt multi-scale GWR to investigate different behavior of COVID-19 incidence in response to the selected socioeconomic and environmental characteristics. Their results report that spatial heterogeneity is strong in the northeastern US region, especially in the New York, Connecticut, and New Jersey states [15]. These studies demonstrate that some areas suffer from the COVID-19 pandemic more than others, and there are clear spatial clusters and great spatial non-stationarity within the US.

Though the previous research has provided new insight into health disparities in COVID-19 in the US, most, if not all, prior studies analyze the *cumulative* numbers of COVID-19 cases or deaths as of a particular date. We argue that this approach overlooks the daily changes in these outcomes and fails to consider the temporal trend of how the pandemic evolves in an area [16]. Some scholars have included the number of days since the first case in their analysis [3, 12]; nonetheless, this approach still does not consider the daily variation. From the epidemiological perspective, an infectious disease epidemic is a consequence of intensive contact with those who have the disease and being exposed to other risk factors [17]. The commonly used cumulative case/death rates in extant literature cannot distinguish different patterns of transmission over the same time period because epidemic frequency and occurrence may vary in two regions having the same incidence rate during the given period [16].

In this study, we fill the gap by explicitly measuring three temporal dimensions of the COVID-19 pandemic in US counties from late-January to mid-September, 2020, namely probability of COVID-19 occurrence, duration of COVID-19 infection, and intensity of COVID-19 transmission. These dimensions allow us to define the long-term risk of COVID-19 infection in US counties. We explore the spatial distributions of different levels of risk, and then investigate the determinants of the risk level with a geographically weighted ordinal logistic regression model (GWOLR). To the best of our knowledge, the GWOLR model has not been utilized in COVID-19 literature and this analytic approach helps us to not only identify various factors of COVID-19 infection risk but also examine if these impacts are stable over space. The next two sections will discuss the data and methods used in this study, followed by the results section. We will then discuss the findings and draw conclusions from the results.

## Materials

### Data sources

We assembled a dataset of 3,106 counties in the contiguous US with the following data sources: the Coronavirus Live Map [18], County Health Ranking and Roadmaps (CHRR) [19], the Area Health Resources Files [20] and Census Bureau GIS data [21]. It should be noted that the CHRR data synthesize various socioeconomic and health variables from several national datasets, such as the American Community Survey, Census Population Estimates, Small Area Income and Poverty Estimates, and the National Center for Health Statistics.

### Measures

Our dependent variable is an ordinal variable comprising of four categories: low-risk, mild-risk, moderate-risk, and high-risk counties. These categories are created with the following three epidemiological measures, namely a frequency index (*α*) that measures the occurrence probability, a duration index (*β*) that describes the persistence of transmission, and an intensity index (*γ*) that quantifies the epidemic severity. We first obtained the daily number of confirmed COVID-19 cases in a county between January 22 and September 11, 2020, and these numbers are based on the information released by the Centers for Disease Control and Prevention, state- and local-level public health agencies [18]. We then followed the work by Wen et al. [16] to transform the daily COVID-19 counts into the above three epidemiological risk indices for each county. The details about how these indices are calculated can be found in the S1 Appendix.

We applied the local indicator of spatial association (LISA) technique [22] to each epidemiological measure to identify spatial patterns, including clustering and outliers. The LISA index is defined as

$$I_{i}=\frac{X_{i}-\bar{X}}{S}\times \sum_{j=1}^{3106}W_{ij}\times \frac{X_{j}-\bar{X}}{S}$$

where *I*_*i*_ is the LISA index for county *i*, *X*_*i*_ and *X*_*j*_ are respectively the values of an epidemiological measure for county *i* and *j* (*j*≠*i*), $\bar{X}$ is the mean value of the measure across all counties, *S* represents the standard deviation of the epidemiological measure, and *W*_*ij*_ is the first-order queen contiguity-based spatial weights matrix. We obtained the results of the “hotpot” status of each county and recoded if a county falls into the so-called “hotspots” of each measure (i.e., a county with a high value of probability of occurrence is surrounded by counties with similar levels of risk) or is surrounded by counties with high risks. As such, we defined low-risk counties as those that do not fall into any hotspot, and high-risk counties indicate those counties in a hotspot for all three epidemiological measures. Similarly, mild-risk counties refer to those falling into a hotpot for only one measure, and moderate-risk counties are those falling into a hotspot for any two measures. We have conducted sensitivity tests with other weighting schemes (e.g., rook contiguity) and found that the results are largely the same.

Our independent variables can be categorized into four groups: (1) demographic composition and the number of days since the first confirmed case, (2) labor market factors, (3) housing inequalities, and (4) health infrastructure, and we discussed them as follows.

Demographic composition includes the percentages of non-Hispanic blacks (hereinafter blacks), non-Hispanic Asians (hereinafter Asians), Hispanics, and population density (dividing population by county land area) which is standardized to avoid singularity. We included the number of days between the first confirmed case and September 11, 2020.

We used five variables to assess the labor market of a county: the percentage of population aged 65 and older, unemployment rate, logged median family income, the percentage of essential workers, and the percentage of workers who work outside the county of residence.

Housing inequalities are gauged with the percentage of housing units with severe housing problems (e.g., lack of complete kitchen or plumbing facilities), the ratio of the 80^th^ and 20^th^ percentiles of income, and the nonwhite/white segregation index (i.e., dissimilarity index). The segregation index ranges from 0 to 100 and higher values indicate higher levels of residential segregation between nonwhite and white residents [23].

Health infrastructure includes the percentage of adult population without health insurance and the Health Professional Shortage Area (HPSA). The HPSA coding scheme is included in the Area Health Resources Files and has three levels: counties that are not at any shortage, part of the county is at shortage, and the whole county is at shortage. A conventional approach is to create two dummy variables in the analysis; however, we treated this ordered variable with the ridit method [24]. This technique assigns numerical scores to each category based on the distribution of this ordered variable and helps users to account for the ordinality in various disciplines [25].

## Methodology

### Analytic strategy

Using Federal Information Processing Series (FIPS) codes, we merge the data to the county-level shapefile from US Census Bureau [26] using ArcGIS [27]. We first visualize the spatial distribution of different levels of COVID-19 risk. We then conduct descriptive statistics analysis for all counties and by levels of risk and then implemented pair-wise comparison test to better understand the differences across groups. As our dependent variable is an ordinary variable, we implement the conventional (i.e., non-spatial) ordinal logistic analysis to assess the overall associations between COVID-19 risk and the covariates. Furthermore, to investigate the potential spatially non-stationary associations and given the ordinal nature of the outcome variable, we apply the geographically weighted ordinal logistic regression (GWOLR) technique to our data and use the Monte Carlo test to formally examine whether the associations of interest vary across US counties. Regarding model specification, we implement four nested regression models as follows. Model 1 includes the demographic composition and days since the first confirmed case and Model 2 further considers labor market factors. We add housing inequality variables to Model 3 and health infrastructure covariates to Model 4, respectively. Both non-spatial modeling and GWOLR will be applied to these model specifications and we will compare the results with various model fit diagnostics (e.g., concordance index and correction rate). All the analyses are implemented in R [28].

### Geographically weighted ordinal logistic regression

Let *Y*_*i*_ be the categorical dependent variable with four ordered risk levels (1 = low-risk, 2 = mild-risk, 3 = moderate-risk, 4 = high-risk) at county *i*, and ***x***_*i*_ be the selected row-vector of the county-level characteristics. A conventional/non-spatial global ordinal logistic regression model (OLR) describes the relationship between *Y*_*i*_ and ***x***_*i*_ via logit equations:

$$logit[P(Y_{i}\leq j)]=\log\frac{P(Y_{i}\leq j)}{1-P(Y_{i}\leq j)}=a_{j}-x_{i}\beta$$ (1)

where $P(Y_{i}\leq j)=\frac{exp(a_{j}-x_{i}\beta )}{1+exp(a_{j}-x_{i}\beta )}$ is the cumulative probability for outcome category *j* (*j* = 1,2,3), and *a*_*j*_ represents separate intercept parameter for each logit; ***β*** is the vector of regression coefficients that are constant across logits. A positive coefficient implies the increasing probability of being in higher COVID-19 risk categories. The probability of being in category *j* can be computed by taking differences between the cumulative probabilities,

$$P(Y_{i}=j)=P(Y_{i}\leq j)-P(Y_{i}\leq j-1)\;\mathrm{for}\;j=2,3$$

and *P*(*Y*_*i*_ = 1) = *P*(*Y*_*i*_≤1). In fitting model (1) to data, all parameters can be estimated with maximum likelihood methods simultaneously.

The OLR in (1) builds the model specification without a spatial perspective and is unable to explore the potential spatially varying relationships between the dependent and independent variables. As such, GWOLR developed by Dong et al. [29] is a spatial local modeling technique that extends OLR to the GWR modeling framework and allows regression coefficients to vary across space. As such, the model can be expressed as:

$$logit[P(Y_{i}\leq j)]=\log\frac{P(Y_{i}\leq j)}{1-P(Y_{i}\leq j)}=a_{j}(u_{i},v_{i})-x_{i}\beta (u_{i},v_{i})$$

where (*u*_*i*_, *v*_*i*_) represents the geographical coordinates of county *i*.

The regression coefficients in GWOLR can be estimated by a geographically weighted local likelihood approach and carried out with the maximum likelihood method. The principle is to place a kernel around a county, and then compute the local estimates using all the observations within the kernel window. Several choices exist for the kernel function; see Fotheringham et al. [30] for more discussions. In this study we use the commonly used adaptive bisquare kernel function given below:

$$w_{ij}=\left\{ \begin{array}{l} \;\left[1-{\left(\frac{d_{ij}}{h}\right)}^{2}\right]\\ 0\;\mathrm{otherwise} \end{array}\right.\;if\;d_{ij}<h$$

where w_*ij*_ is the weight value of county *j* for the coefficient estimation in county *i* and *d*_*ij*_ is the distance between county *i* and county *j*. As can be seen, counties closer to the county *i* are assigned larger weights than those farther away. The parameter *h* is the kernel bandwidth regulating the kernel size, and finding an optimal one is crucial to GWOLR estimations. Following Dong et al. [29], we estimate the optimal bandwidth by minimizing the cross validation (CV) criteria

$$CV=\sum_{i=1}^{n}{\left(1-{\left|\hat{P}(Y_{i}=j)\right|}^{k}\right)}^{2}$$

for *k* being a binary indicator variable with *k* = 1 when *Y*_*i*_ = *j* and *k* = 0 otherwise.

The model calibrations and estimations for GWOLR are carried out with the R library developed by Dong et al. [29], which are available in the Supporting Information of Dong et al’s work.

### Model evaluations

To validate the use of GWOLR, we calculate four metrics to compare between the four local models and for comparisons with the global OLR. These criteria include the pseudo R^2^ derived using multiple correlation as suggested in Agresti and Tarantola [31], the deviance, the overall proportion of correct classification (i.e., correction rate), and the concordance index based on the area under the Receiver Operating Characteristic (ROC) curve as defined by Hand and Till [32]. The smaller the deviance and the higher the other three statistics, the better the model predicts the ordinal outcome using model fit [25, 31].

### Test of spatial non-stationarity

Investigation on whether a particular regression coefficient varies across space is essential for this study and it takes a formal statistical test to attain this purpose [29]. Therefore, a Monte Carlo randomization test approach is used to examine whether the local coefficients vary significantly across space, which is known as spatial non-stationarity. Even though this technique is computationally demanding, it does not need to derive the sampling distribution of the variance of the local parameter estimates, an advantage over other parametric techniques. The concept is to test the null hypothesis that the local coefficients drawn from the GWOLR models do not vary greatly enough (i.e., statistically constant) using the permuted data with locations arbitrarily reallocated in space. If the variance of the local parameter estimates of the original model fall in the 5% tails of those obtained from the simulated models, we have evidence to reject the null hypothesis and to conclude that non-stationarity exists in the local regression estimates.

## Results

### Descriptive analysis results

**Fig 1:**
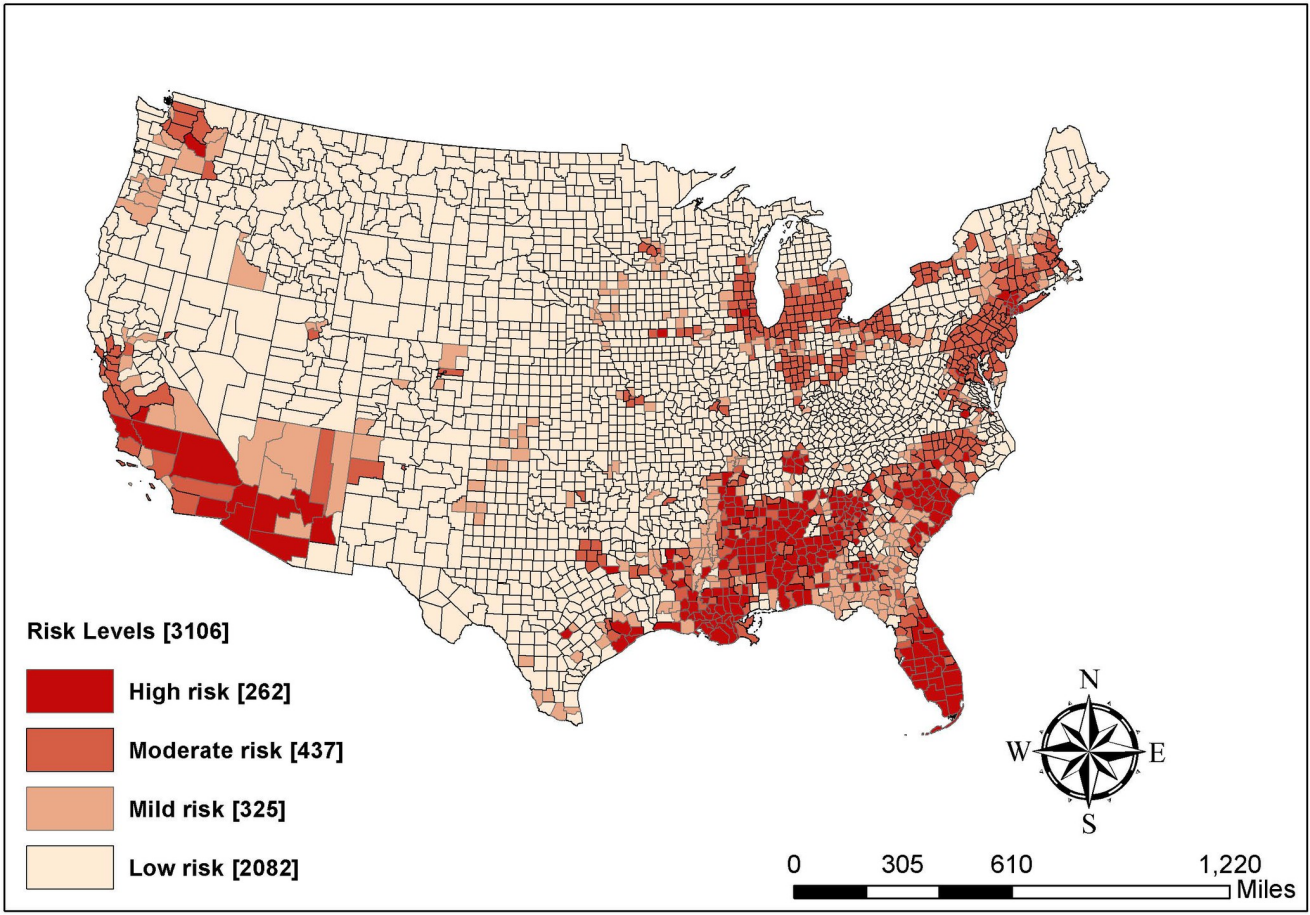
Levels of COVID-19 risks in US counties. Source: The figure is drawn using ArcGIS software. The shapefile used to create the map is from the US Census Bureau and therefore reproducible by law.

Fig 1 demonstrates the spatial distribution of different levels of COVID-19 risk classified based on LISA hotspot analysis (see S1 Appendix for LISA maps). To be specific, approximately 8 percent of the contiguous counties (n = 262) are categorized into the high-risk group and these counties are clustered in the following areas: southern California and Arizona (the US-Mexico border), the Black Belt, New York City, and southern Florida. Almost 15 percent of counties (n = 437) are found to have a moderate risk and they are concentrated in the northeastern region (especially New York, Connecticut, and New Jersey) and the Great Lakes region. About 10 percent of counties show a mild-risk of COVID-19 infection (n = 325) and they are in the States of Washington and Arizona and southern Georgia and South Carolina. Slightly more than two-thirds of counties (n = 2,082) are in the low-risk group. These patterns echo the extant literature suggesting that the COVID-19 risk is not evenly distributed in the US [12, 15].

County characteristics stratified by COVID-19 risk level and the results of pairwise t-tests are presented in Table 1. We summarize a few important observations. First, the percentages of blacks and Asians are found to be higher in mild/moderate/high COVID-19 risk counties, compared to low-risk counties. For example, while only 4.4% of residents are blacks in low-risk counties, more than one-fourth of residents are blacks (26.3%) in high-risk counties. It is also likely that Asians are found to live in moderate/high-risk counties, rather than low/mild-risk counties. Proportion of Hispanics in county is not statistically different across the level of COVID-19 risk. Population density as well as the days since the first confirmed case is likely to be higher in moderate/high-risk counties, compared to that of low/mild-risk counties.

**Table 1:** Descriptive statistics of all variables by COVID-19 risk.

	Overall		Low risk		Mild risk		Moderate risk		High risk		
Variables	Mean or %	S.D.	Mean or %	S.D.	Mean or %	S.D.	Mean or %	S.D.	Mean or %	S.D.	t-test results^†^
% Blacks	9.1	14.4	4.4	8.6	17.4	19.3	14.9	15.6	26.3	19.7	a, b, c, e, f
% Asians	1.5	2.5	1.0	1.2	1.7	2.6	3.2	4.8	2.0	3.0	a, b, c, d, f
% Hispanics	9.7	13.9	9.6	14.6	10.8	15.4	9.1	8.9	10.4	13.4	
Population density (logged)	3.8	1.8	3.3	1.6	4.2	1.5	5.5	1.5	4.8	1.6	a, b, c, d, e, f
Time to first case (days)	88.3	24.5	82.5	27.0	95.6	12.8	102.4	10.3	101.6	8.4	a, b, c, d, e
% Older than 65	19.3	4.7	20.1	4.6	18.1	3.9	17.3	4.1	17.7	4.7	a, b, c, d
% Unemployed	4.1	1.4	4.0	1.4	4.4	1.6	4.1	1.0	4.5	1.6	a, c, d, f
Median income (standardized)	0.0	1.0	-0.1	0.8	-0.1	1.1	0.7	1.4	-0.1	1.2	b, d, f
% Essential workers	68.1	6.7	68.5	5.9	69.0	6.8	65.1	8.7	69.1	7.1	b, d, f
% Work outside the county of residence	30.8	17.8	28.5	17.3	33.3	17.6	35.9	17.0	37.1	19.4	a b, c, d, e
% Severe housing problems	14.4	4.4	13.5	4.0	15.6	4.4	16.1	4.1	17.2	4.8	a, b, c, e, f
Nonwhite-white segregation index	30.8	12.4	29.4	12.2	31.6	12.3	35.9	12.1	33.0	12.4	a, b, c, d, f
Income ratio (80^th^ to 20^th^)	4.5	0.8	4.4	0.7	4.7	0.9	4.5	0.8	5.0	0.9	a, b, c, d, e, f
% Uninsured	11.4	5.1	11.5	5.2	12.0	5.2	9.7	4.5	13.4	4.0	b, c, d, e, f
HPSA											
No shortage	10.6		10.5		10.3		13.5		6.1		c, f
Part of the county is at shortage	62.9		62.3		61.2		69.1		60.3		b, d, f
The whole county is at shortage	26.5		27.2		28.3		17.4		33.6		b, c, d, f
N	3,106 (100.0%)		2,082 (67.0%)		325 (10.5%)		437 (14.1%)		262 (8.4%)		

^†^ Two sample t-test results (at least 0.05 significance level

a: low risk vs. mild risk

b: low risk vs. moderate risk

c: low risk vs. high risk

d: mild risk vs. moderate risk

e: mild risk vs. high risk

f: moderate risk vs. high risk)

Second, the percentages of the unemployed and people working outside the county of residence are higher in high COVID-19 risk counties than those of counties where COVID-19 risk is low, mild, or moderate. The proportion of senior residents who are older than 65 is higher in low-risk counties, compared to that of higher risk counties. In moderate-risk counties, median income is shown to be highest, and the proportion of essential workers is found to be lowest.

Third, regarding housing inequality, moderate/high COVID-19 risk counties are more likely to have severe housing problems (17.2% in high-risk counties) and nonwhite-white segregation issues (35.9% in moderate-risk counties). Income inequality, measured by income ratio (80^th^ to 20^th^), is the highest in high COVID-19 risk counties.

Fourth, high COVID-19 risk counties appear to have poor health infrastructure. Highest proportion of uninsured residents (13.4%) are found to reside in high-risk counties, and about one-third of high-risk counties (33.6%) are entirely a Health Professional Shortage Area (HPSA), and only about 6% of high-risk counties have no shortage problems.

### Global OLR results

In Table 2, we conducted ordinal logistic regression analyses to examine what characteristics of county are associated with the level of COVID-19 risk. In Model 1, the relationships between demographic characteristics and the level of COVID-19 risk in county are presented. One percent point increase in black residents in a county is associated with a 6% increase in the odds of moving up to the next COVID-19 risk level (i.e., low to mild, mild to moderate, moderate to high; OR = exp (0.059) = 1.06). Similarly, an increase in the Hispanic population is marginally correlated with higher COVID-19 risk in county (OR = exp (0.010) = 1.01). The proportion of Asians, however, does not predict the level of COVID-19 risk in county. One unit increase in population density (logged) is related to 48% (OR = exp (0.389) = 1.48) increase in the odds of changing to the higher COVID-19 risk level in county. The number of days since the first confirmed case is positively associated with COVID-19 risk level, with an about 4% (OR = exp (0.043) = 1.04) increase in the odds of higher risk for every one-day increase.

**Table 2:** Ordinal logistic regression for COVID-19 risk (N = 3,106).

	Model 1		Model 2		Model 3		Model 4	
	Odds Ratio	95% CI	Odds Ratio	95% CI	Odds Ratio	95% CI	Odds Ratio	95% CI
**Demographics & Time**								
% Blacks	1.06***	(1.05–1.07)	1.07***	(1.06–1.08)	1.07***	(1.06–1.07)	1.06***	(1.05–1.07)
% Asians	0.99	(0.96–1.02)	0.97	(0.94–1.00)	0.96*	(0.92–1.00)	0.96*	(0.92–0.99)
% Hispanics	1.01**	(1.00–1.02)	1.01**	(1.00–1.02)	1.01**	(1.00–1.02)	1.00	(0.99–1.01)
Population density (logged)	1.48***	(1.37–1.59)	1.50***	(1.38–1.62)	1.40***	(1.29–1.53)	1.47***	(1.34–1.60)
Time to first case	1.04***	(1.03–1.05)	1.05***	(1.04–1.06)	1.05***	(1.04–1.06)	1.05***	(1.04–1.06)
**Labor Market Factors**								
% Older than 65			1.01	(0.99–1.03)	1.01	(0.99–1.04)	1.01	(0.99–1.03)
% Unemployed			1.07	(0.99–1.16)	1.01	(0.93–1.09)	1.07	(0.98–1.17)
Median income (standardized)			1.75***	(1.52–2.01)	1.99***	(1.70–2.32)	2.19***	(1.87–2.56)
% Essential workers			1.07***	(1.05–1.09)	1.08***	(1.06–1.10)	1.08***	(1.05–1.10)
% Work outside the county of residence			1.02***	(1.01–1.02)	1.02***	(1.01–1.03)	1.02***	(1.01–1.02)
**Housing Inequalities**								
% Severe housing problems					1.01	(0.99–1.04)	1.01	(0.98–1.04)
Nonwhite-white segregation index					1.02***	(1.01–1.03)	1.02***	(1.02–1.03)
Income ratio (80^th^ to 20^th^)					1.30***	(1.12–1.51)	1.26**	(1.09–1.47)
**Health Infrastructure**								
% Uninsured							1.06***	(1.03–1.09)
HPSA (ridit scores)							1.73**	(1.14–2.61)
Cut 1	7.07***	(6.23–7.91)	13.22***	(11.32–15.12)	15.44***	(13.26–17.62)	16.28***	(14.08–18.48)
Cut 2	7.86***	(7.04–8.68)	14.05***	(12.13–15.97)	16.29***	(14.09–18.49)	17.13***	(14.92–19.34)
Cut 3	9.38***	(8.52–10.24)	15.63***	(13.69–17.57)	17.89***	(15.68–20.10)	18.75***	(16.52–20.98)

Model 2 includes labor market factors of county, in addition to the variables used in Model 1. A one-percentage-point increase in the essential workers is associated with 8% increase in the odds of changing to the higher COVID-19 risk level. In terms of standardized median income, a one-unit increase in this variable elevates the odds of having a higher COVID-19 risk level by 75% (OR = exp (0.559) = 1.75) in a county. Both the percentage of unemployment and the proportion of people older than 65 are not statistically related to the level of COVID-19 risk in county.

Model 3 additionally estimates the effects of factors indicating housing inequalities. One unit increase in nonwhite-white segregation index is associated with increased odds of changing to the higher COVID-19 risk level by 2%, whereas severe housing problem is not significant to the level of COVID-19 risk level in county. The positive relationships between variables measuring housing inequalities and COVID-19 risk level in county do not change when additional variables are accounted for in Model 4.

We further add county variables measuring health infrastructure in Model 4. One percentage point increase in the uninsured is associated with a 6% (OR = exp (0.057) = 1.06) increase in the odds of moving up to the next COVID-19 risk level in a county. Also, for one-category increase in HPSA, the odds of having higher COVID-19 risk level increases by 73% (OR = exp (0.546) = 1.73).

It should be noted that, when controlling for the health infrastructure factors, while percent of blacks remains a significant predictor of higher COVID-19 risk in county, percent of Hispanics is no longer statistically significant, suggesting that counties with high concentrations of Hispanics may also have poor health infrastructure. By contrast, the percentage of Asians becomes a significant factor that reduces the COVID-19 risk level after housing inequality variables are considered (i.e., Models 3 and 4). This finding suggests that segregation and income inequality may suppress the relationship between the percentage of Asians and COVID-19 risk.

With the conventional ordinal logistic regression, we found that county’s demographic (i.e., % blacks and population density), the number of days since the first confirmed case, labor market factors (i.e., median income, % essential workers, and % work outside the county of residence), housing inequalities (i.e., % nonwhite-white segregation index, and income ratio), and health infrastructure (i.e., % uninsured and HPSA) are consistently associated with the odds of having higher COVID-19 risk in a county.

### GWOLR results

Following the suggested practice of GWR [30], we present the five number summary of the GWOLR results in Table 3 and visualize the local estimates into maps. The results in Table 3 are based on the specification of Model 4 (Table 2) and an adaptive bisquare kernel weighting scheme with the optimal bandwidth of 847 nearest neighbors (see Table 4). The last column in Table 3 shows the Monte Carlo test results for spatial non-stationarity. It should be noted that we also estimate the model by using a fixed gaussian kernel approach with a distance bandwidth of 4.20 miles; the results of model comparison (details not shown) do not favor the use of fixed kernel over adaptive kernel. We draw the key findings from the GWOLR results as follows. First, all covariates included in the analysis show a spatially non-stationary association with the COVID-19 risk levels based on the Monte Carlo results. That is, the same change in a certain covariate is likely to provoke different changes in the COVID-19 risk. Take racial/ethnic percentages for example, a one-unit change in the percentage of blacks is estimated to have as high as 20 percent increase (OR = exp(0.1841) = 1.20) in the odds of having a higher COVID-19 risk level, but this association is estimated to be negative (i.e., reducing the risk) in some counties (see the minimum number, -0.1974). The same patterns can be found in the percentages of Asians and Hispanics.

**Table 3:** GWOLR estimates and Monte Carlo non-stationarity test results.

	Min.	Q1	Median	Q3	Max	Monte Carlo Test *p*-value
**Demographics & Time**						
% Blacks	-0.1974	0.0451	0.0680	0.0975	0.1841	0.00
% Asians	-0.2720	-0.0687	-0.0227	0.0294	0.3420	0.00
% Hispanics	-0.1145	0.0116	0.0375	0.0793	0.1288	0.00
Population density (logged)	-0.5528	-0.0150	0.3364	0.6106	1.7743	0.00
Time to first case	-0.0146	0.0160	0.0312	0.0659	0.0922	0.00
**Labor Market Factors**						
% Older than 65	-0.1284	-0.0759	-0.0086	0.0413	0.1280	0.00
% Unemployed	-0.8482	-0.3846	-0.1517	0.0493	0.5053	0.00
Median income (standardized)	-0.2730	0.7214	0.9132	1.1062	2.0823	0.00
% Essential workers	-0.0762	0.0194	0.0538	0.0880	0.1646	0.00
% Work outside the county of residence	-0.0143	0.0060	0.0110	0.0181	0.0525	0.02
**Housing Inequalities**						
% Severe housing problems	-0.1288	-0.0481	-0.0138	0.0389	0.1507	0.02
Nonwhite-white segregation index	-0.0181	-0.0031	0.0207	0.0370	0.0841	0.00
Income ratio (80^th^ to 20^th^)	-1.6040	-0.2816	0.0766	0.3928	0.8701	0.00
**Health Infrastructure**						
% Uninsured	-0.1778	-0.0338	0.0376	0.1196	0.3150	0.00
HPSA (ridit scores)	-0.8253	-0.0954	0.5753	1.2363	2.6535	0.01
Cut 1	-1.0240	8.2380	11.4480	15.2780	25.9360	0.00
Cut 2	0.8043	9.4207	13.0645	16.2937	26.7128	0.00
Cut 3	2.9750	11.5070	15.7340	19.4160	32.5380	0.00

**Table 4:** Model comparisons between OLR and GWOLR.

	Model 1		Model 2		Model 3		Model 4	
	global	local	global	local	global	local	global	local
Bandwidth	--	687	--	825	--	815	--	847
Residual deviance	4859.012	3821.575	4699.837	3600.400	4653.724	3502.485	4623.923	3470.945
Pseudo R^2^	0.3893	0.5508	0.4300	0.5953	0.4433	0.6145	0.4492	0.6236
Correction rate	0.6983	0.7592	0.7038	0.7708	0.7067	0.7746	0.7073	0.7788
Concordance index	0.6257	0.7131	0.6496	0.7416	0.6570	0.7512	0.6726	0.7617

**Fig 2:**
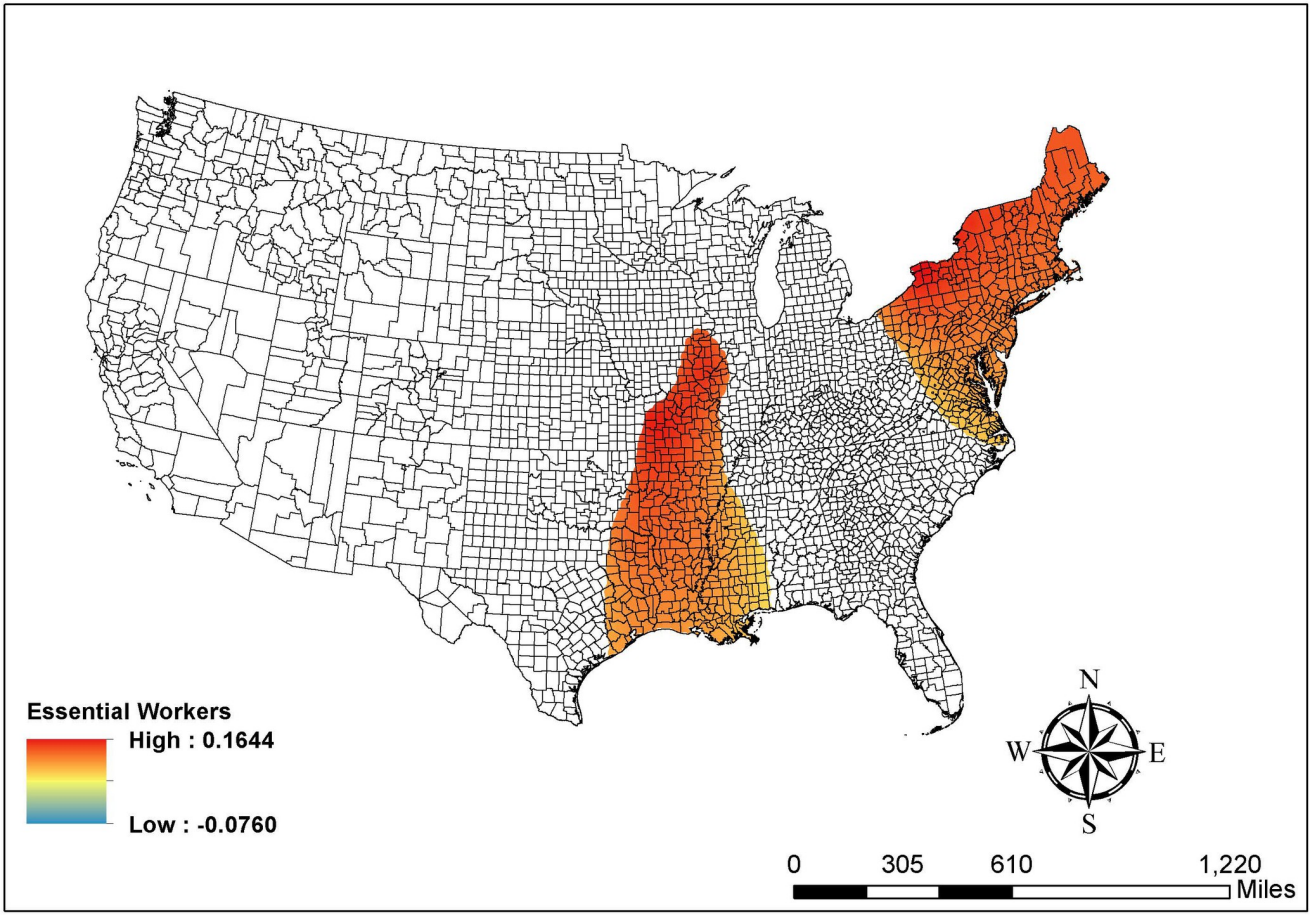
Map of the GWOLR parameter estimates in % essential workers. Source: The figure is drawn using ArcGIS software. The shapefile used to create the map is from the US Census Bureau and therefore reproducible by law.

Second, the five number summary does not consider the local significance test results. To better demonstrate the local patterns, we follow the mapping technique proposed by Matthews and Yang [33] to visualize the percentage of essential workers, percentage of workers working outside the county of residence, and income ratio (maps of other covariates are available upon request). As shown in Fig 2, the positive associations between the percentage of essential workers and the COVID-19 risk levels are statistically significant in the northeastern region and counties along the Mississippi Valley. Counties in these areas mainly drive the positive and significant global association found in Model 4 (coefficient = 0.074, p < .001) and other counties do not have a significant association.

**Fig 3:**
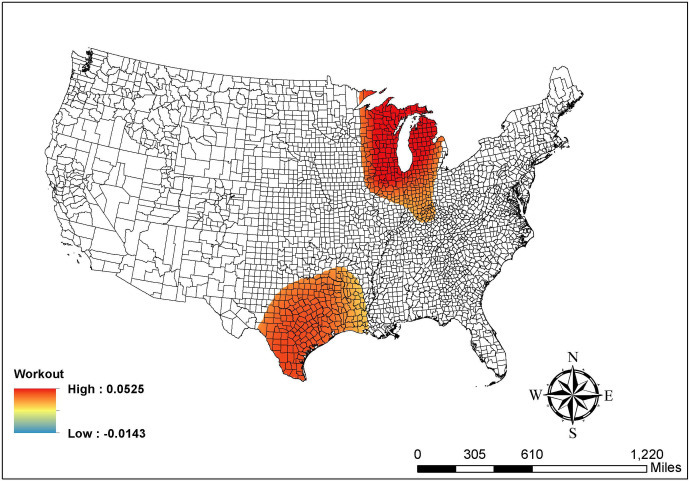
Map of the GWOLR parameter estimates in % working outside the county of residence. Source: The figure is drawn using ArcGIS software. The shapefile used to create the map is from the US Census Bureau and therefore reproducible by law.

**Fig 4:**
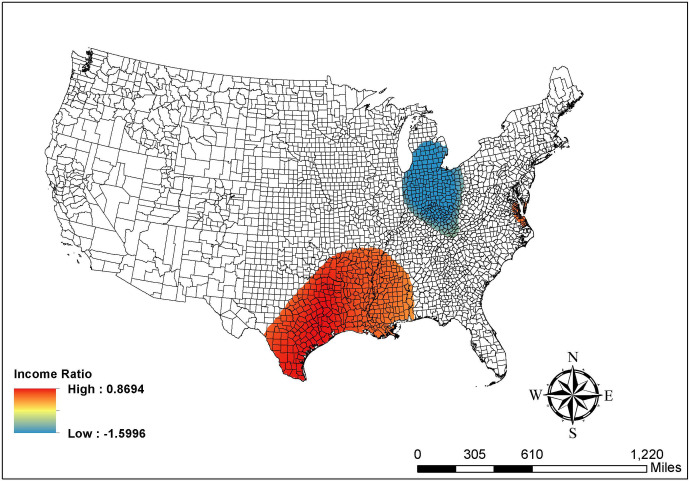
Map of the GWOLR parameter estimates in income ratio. Source: The figure is drawn using ArcGIS software. The shapefile used to create the map is from the US Census Bureau and therefore reproducible by law.

Similarly, Fig 3 indicates that the positive association between the percentage of workers working outside the county of residence and the COVID-19 risk level is only significant among counties in the states next to Lake of Michigan, southern and eastern Texas, and western Louisiana. All these counties show a positive association, which echoes the global finding in Table 2. Furthermore, Fig 4 shows a complex pattern of income ratio. Specifically, many counties in Michigan, Indiana, and Ohio have a negative relationship between income ratio and the COVID-19 risk levels. That is, higher income ratios are associated with lower risk in these blue areas. By contrast, counties in southern and western Texas, Louisiana, Arkansas, and Mississippi report a positive association between income ratio and the COVID-19 risk level (red areas). Fig 4 clearly shows the spatial non-stationarity in local coefficient estimates, which cannot be obtained from the conventional/global ordinal logistic regression models.

We compare the global models with the GWOLR models using various diagnostic statistics and the results are summarized in Table 4. As suggested by these statistics, GWOLR outperforms the global model and this conclusion holds regardless of model specifications. For example, residual deviance is smaller for GWOLR models than for the global ones, and the correction rate is consistently higher among the GWOLR models than their global counterparts. Importantly, the model fit diagnostics persistently improve from Model 1 to Model 4, indicating that the covariates included in our analysis are at least statistically meaningful. As such, we have evidence to conclude that the GWOLR approach better fit the data than the global models and the spatially non-stationary associations provide important implications for existent literature.

## Discussion and conclusion

This study adopts three different temporal indices proposed by Wen et al. [16] to describe the epidemiological characteristics of COVID-19 infection risk in the early stage of the pandemic in US counties. This application allows us to identify areas with four different levels of risk drawn from the local spatial autocorrelation. We investigate the factors associated with infection risks with both global and local ordinal logistic modeling techniques. To our knowledge, this study is the first application of the GWOLR modeling in the rapidly growing COVID-19 literature and our findings demonstrate great spatial variability in the associations between COVID-19 risk level and covariates. As most existing studies rely on cumulative case/death counts or rates, we offer a new perspective to take into account different temporal dimensions of the pandemic process over a certain period of time.

Compared with the extant literature [8, 12, 13, 15], our findings based on the global models confirm that minority concentrations, especially blacks, and absolute socioeconomic conditions are important factors that may heighten COVID-19 infection risk over time. However, several findings indicate that the determinants of risk level may differ from those of COVID-19 incidence rates. For example, the association between percent of Hispanics and COVID-19 risk level can be explained by health infrastructure measures, which has not been commonly reported in the literature (see [3]). In addition, the role of percentage of uninsured population in a county has been found to be ambiguous [3, 6], but our results show a consistent and positive relationship with the COVID-19 risk. This finding seems to echo the claim that health insurance coverage can directly contribute to the health and economic disparities that already exist in the country [15, 34]. Importantly, we identify that many counties in the Great Lakes region fall into the high- or moderate-Risk categories, which is not often reported in previous research.

Our GWOLR findings advance our understanding of the COVID-19 risk in US counties in at least two ways. On the one hand, our Monte Carlo tests suggest that spatial non-stationarity commonly exists in the relationships between COVID-19 risk level and other covariates. The significant global relationships are likely to be driven by some counties and these relationships tend to differ across space in terms of magnitude and/or direction (i.e., positive or negative). In other words, the previous findings of the global relationship may not hold for all US counties and the same change in a covariate may result in different responses in COVID-19 risk. On the other hand, our ordinal dependent variable is based on three temporal dimensions of the pandemic, which provides a more comprehensive picture of how the pandemic has unfolded in an area than a single measurement, such as COVID-19 incidence or death rate [14, 15]. This approach has not been commonly adopted in the literature and we use the appropriate analytic methods to untangle the relationships between COVID-19 risk level and its covariates.

Our GWOLR findings suggest that a place-based perspective may be more effective in developing potential interventions to lower COVID-19 risk at the county level. For example, providing personal protective equipment to essential workers may be more effective in counties in the northeastern region or southern and eastern Texas as the percentage of essential workers is positively associated with COVID-19 risk level. Similarly, reducing income inequality, the pre-existing structural barriers to health, may be more important for counties in Texas, Arkansas, and Louisiana than for those in other states. The typical one-size-fits-all policy may not be effective, in light of the great spatial non-stationarity unveiled in our analysis.

This study is subject to several limitations. First, our daily data focus on the early stage of the pandemic in the US and the geographical patterns identified in this study may change when the study period is extended. Second, using a different unit of analysis (e.g., census tracts) may alter our conclusions and findings [35]. The daily COVID-19 data at a finer spatial resolution are available for major cities (e.g., New York City), which cannot be used for a nationwide investigation. Third, the availability of COVID-19 tests over the study period is not available, which may affect the daily number of COVID-19 cases and our results. However, it should be noted that our COVID-19 daily data are highly consistent with those from other sources, such as Johns Hopkins University (correlations > 0.99) and the temporal trends (available upon request) are almost identical, suggesting that the quality of COVID-19 data is high and reliable. Finally, this study is ecological and the findings cannot be generalized to other levels (e.g., individuals).

To sum up, this study identifies four different COVID-19 risk levels with three temporal dimensions of the pandemic. This approach offers richer information about how the COVID-19 pandemic evolves over time and allows us to identify the key factors associated with different levels of risk. This perspective is a novel use of spatial risk identification and can be applied to other rapidly spread infectious diseases. While the pandemic has been contained with vaccination, the numbers of new confirmed cases and deaths have grown recently. It becomes critical to focus on local needs for interventions and our study serves as an example of how to identify key factors from a local perspective.

## Supporting information

S1 Appendix Details of three epidemiological indices and supplementary figures. (DOCX)
