# Supplementary material for: Assessing COVID-19 risk with temporal indices and geographically weighted ordinal logistic regression in US counties
Source: PLoS One. 2022 Apr 6;17(4):e0265673. doi: 10.1371/journal.pone.0265673 (PMC8985941; doi:10.1371/journal.pone.0265673)
Supplement: S1 Appendix — (DOCX) [file pone.0265673.s001.docx]

**S1 Appendix: Details of three epidemiological indices and supplementary figures**

*Frequency index (α)*

The frequency index, also called the occurrence probability, measures how frequent COVID-19 cases occur, and is defined as

$$\alpha=\frac{EW}{TW}$$

where EW is the total number of weeks with case occurrence, and TW is the total number of weeks during the study period. The value of α represents the proportion of weeks and ranges from 0 to 1. The more the value approaches 1, the higher the disease probability occurs in certain weeks.

*Duration index (β)*

The duration index describes how long an COVID-19 epidemic prevails. The index is calculated as the average number of weeks an epidemic wave lasted.

$$\beta=\frac{EW}{EV}$$

EW is defined as above. EV is the total number of epidemic waves (epidemic wave is defined as successive weeks with the occurrence of uninterrupted cases). Large value of $\beta$ indicates that cases would less likely to disappear once they occur.

*Intensity index (*$\gamma$*)*

The intensity index assesses how significant the COVID-19 cases occur in consecutive weeks. It assess the epidemic severity of transmission and is derived as the mean incidence rate of cumulative COVID-19 cases occurring in consecutive weeks per epidemic when cases successively occur.

$$\gamma=\frac{IR}{EV}$$

Here, IR denotes the incidence rate of COVID-19 (i.e., total number of confirmed cases divided by total population) in the given period. EV is as defined above. Large values of $\gamma$ refers a time-concentrated transmission.


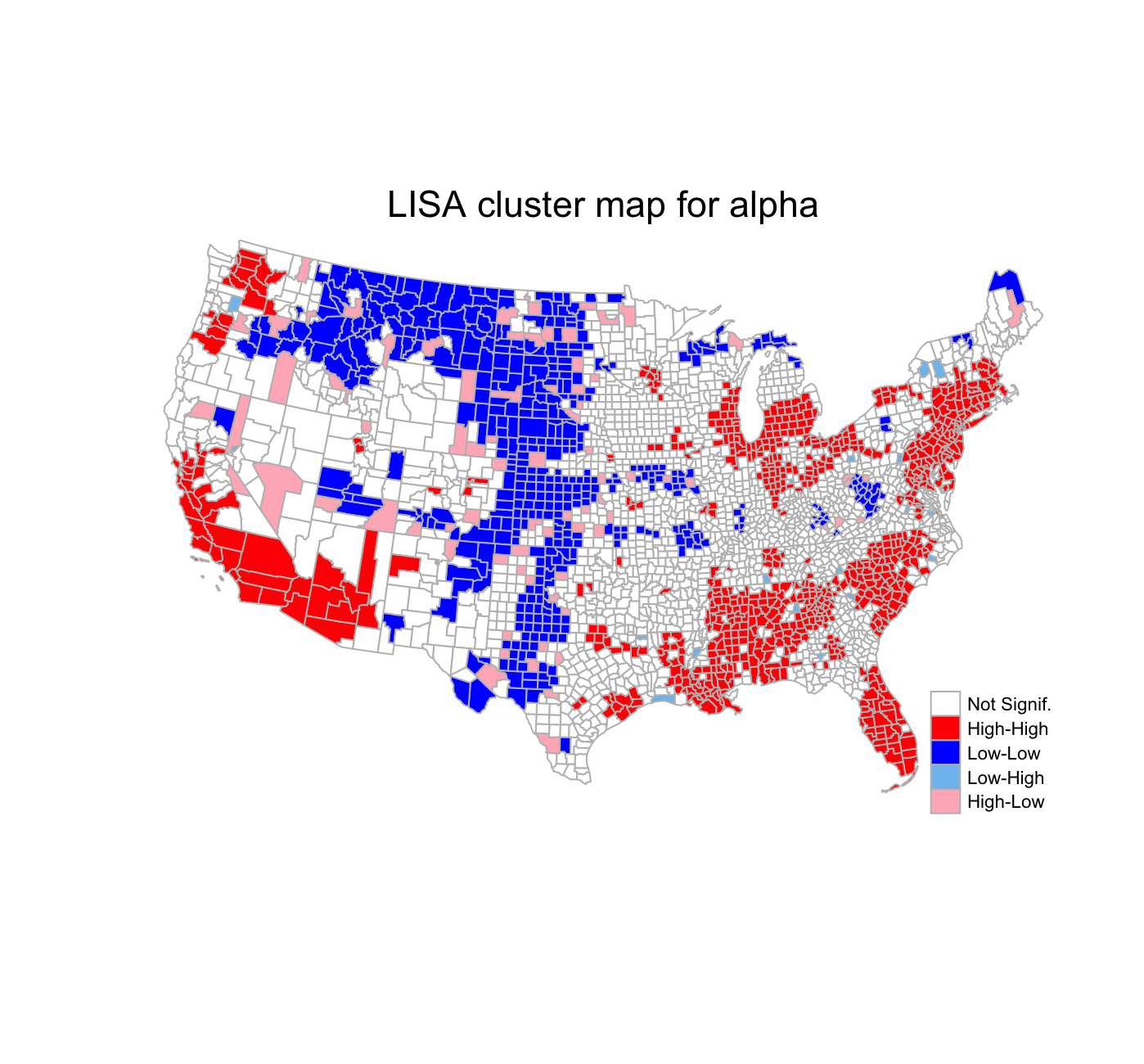


S1 Fig 1. LISA map of occurrence probability (α)


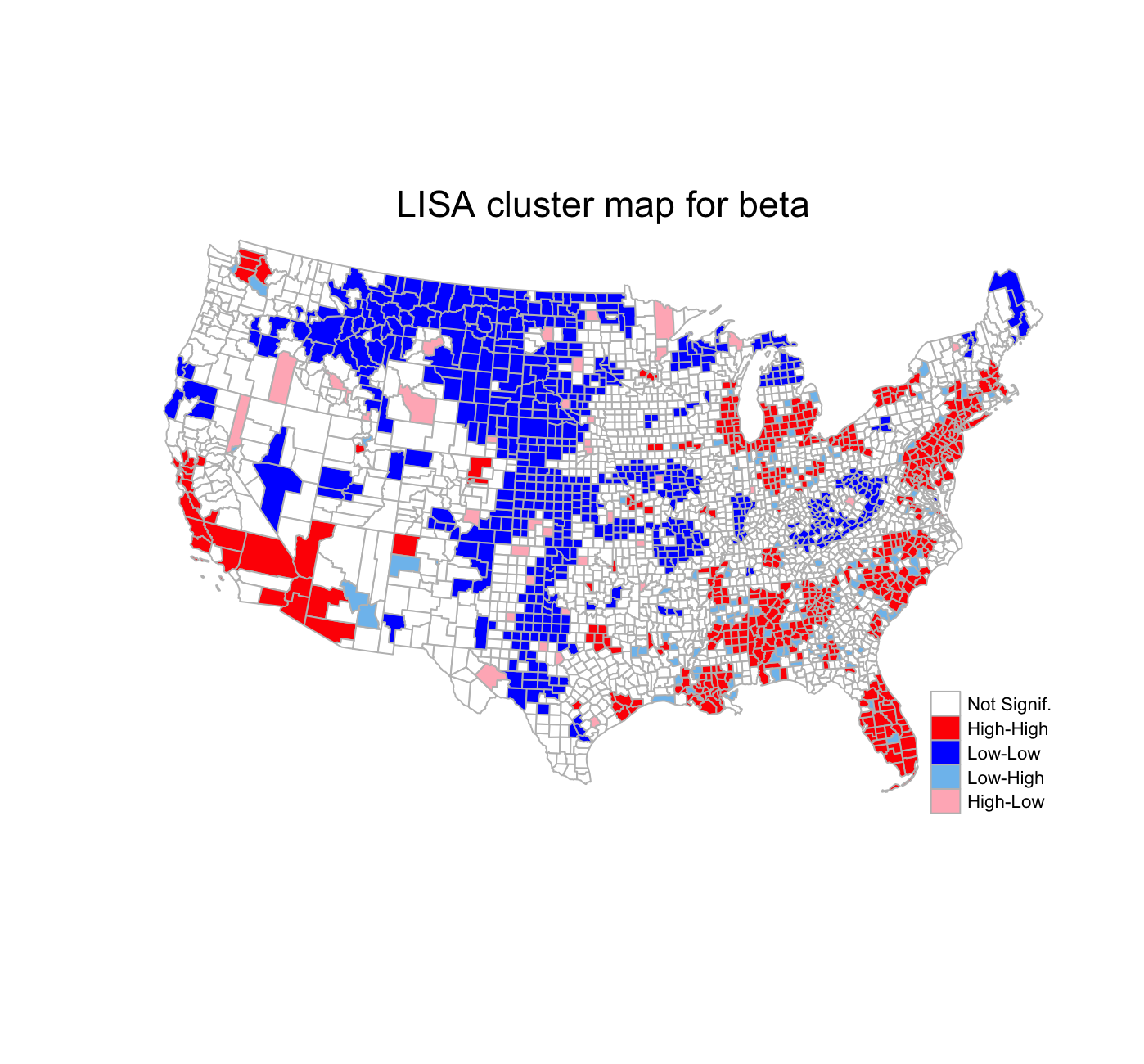


S1 Fig 2. LISA map of duration index (β)


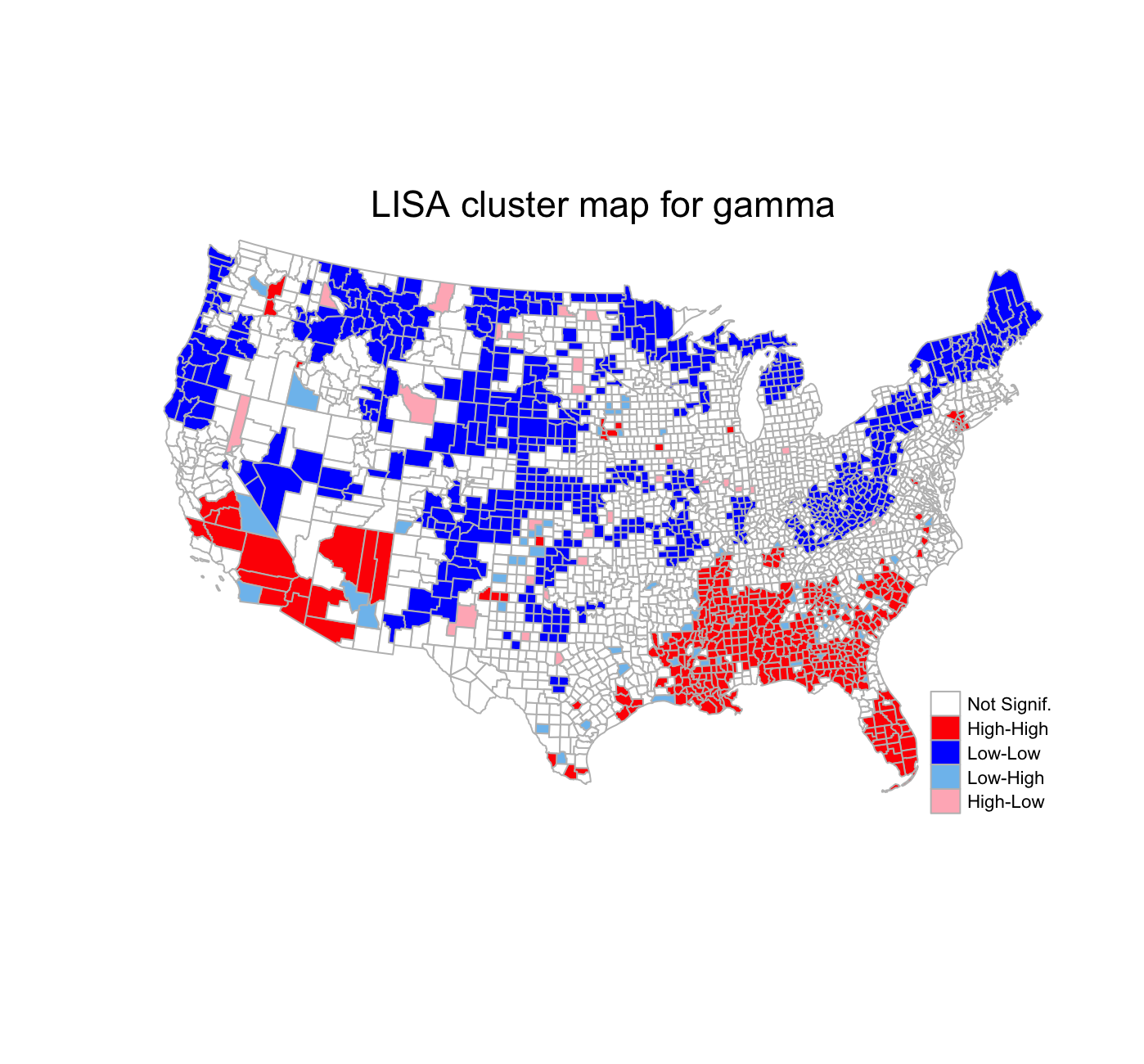


S1 Fig 3. LISA map of intensity index (γ)
